# Effects of Low-Protein Diet on lipid and anthropometric profiles of patients with chronic kidney disease on conservative management

**DOI:** 10.1590/2175-8239-JBN-3842

**Published:** 2018-06-25

**Authors:** Bruna Carvalho Fontes, Juliana Saraiva dos Anjos, Ana Paula Black, Nara Xavier Moreira, Denise Mafra

**Affiliations:** 1 Universidade Federal Fluminense, Programa de Pós-Graduação em Ciências Cardiovasculares, Niterói, RJ, Brasil.; 2 Universidade Federal Fluminense, Programa de Pós-Graduação em Ciências Médicas, Niterói, RJ, Brasil.; 3 Universidade Federal Fluminense, Faculdade de Nutrição, Departamento de Nutrição e Dietética, Niterói, RJ, Brasil.

**Keywords:** Kidney Disease, Chronic, Cardiovascular Diseases, Diet, Protein-Restricted, Dyslipidemias, Overweight, Obesity, Falência Renal Crônica, Dieta com Restrição de Proteínas, Doenças Cardiovasculares, Dislipidemias, Sobrepeso, Obesidade

## Abstract

**Introduction:**

Chronic Kidney disease (CKD) patients have a high prevalence of cardiovascular mortality, and among the risk factors are dyslipidemia and obesity, common findings in the early stages of CKD. The aim of this study was to evaluate the effects of low protein diet (LPD) on the lipid and anthropometric profile in non-dialysis CKD patients.

**Methods:**

Forty CKD patients were studied (20 men, 62.7 ± 15.2 years, glomerular filtration rate (GFR) 26.16 ± 9.4 mL/min/1.73m^2^). LPD (0.6g/kg/d) was prescribed for six months and, biochemical and anthropometric parameters like body mass index (BMI), waist circumference and body fat mass (assessed by dual X-ray absorptiometry - DXA) were evaluated before and after six months with LPD.

**Results:**

After six months of nutritional intervention, patients presented reduction on BMI (from 28.1 ± 5.6 to 27.0 ± 5.3 Kg/m^2^, *p* = 0.001), total cholesterol (from 199.7 ± 57.1 to 176.0 ± 43.6mg/dL, *p* = 0.0001), LDL (from 116.2 ± 48.1 to 97.4 ± 39.1 mg/dL, *p* = 0,001) and uric acid (from 6.8 ± 1.4 to 6.2 ± 1.3 mg/dL, *p* = 0.004). In addition, GFR values were increased from 26.2 ± 9.5 to 28.9 ± 12.7mL/min (*p* = 0.02). The energy, proteins, cholesterol and fiber intake were reduced significantly.

**Conclusion:**

LPD prescribe to non-dialysis CKD patients for six months was able to improve some cardiovascular risk factors as overweight and plasma lipid profile, suggesting that LPD can be also an important tool for protection against cardiovascular diseases in these patients.

## RESUMO

**Introdução:**

Pacientes com Doença Renal Crônica (DRC) possuem alta prevalência de mortalidade cardiovascular e, dentre os fatores de risco, encontram-se alterações no perfil lipídico e excesso de peso, que são achados comuns na DRC. O objetivo deste estudo foi avaliar os efeitos da dieta hipoproteica sobre o perfil antropométrico e lipídico de pacientes com DRC em tratamento conservador.

**Métodos:**

Foram estudados 40 pacientes com DRC (20 homens, 62,7 ± 15,2 anos, e Taxa de Filtração Glomerular (TFG) de 26,2 ± 9,4 mL/min/1,73m^2^). Os pacientes receberam prescrição de dieta hipoproteica (0,6g/kg/d) e parâmetros bioquímicos e antropométricos como índice de massa corporal (IMC), circunferência da cintura (CC) e percentual de gordura corporal (GC) avaliado por absorciometria com raio-x de dupla energia (DXA), foram analisados antes e após 6 meses de intervenção.

**Resultados:**

Os pacientes apresentaram após 6 meses, redução do IMC (de 28,1 ± 5,6 para 27,0 ± 5,3Kg/m^2^, *p* = 0,001), colesterol total (de 199,7 ± 57,1 para 176,0 ± 43,6mg/dL, *p* = 0,0001), LDL (de 116,2 ± 48,1 para 97,4 ± 39,1 mg/dL, *p* = 0,001) e ácido úrico (de 6,8 ± 1,4 para 6,2 ± 1,3 mg/dL, *p* = 0,004) e, aumento da TFG de 26,2 ± 9,5 para 28,9 ± 12,7mL/min (*p* = 0,02). Houve redução significativa na ingestão de energia e proteínas, bem como de colesterol e fibras.

**Conclusão:**

A intervenção com dieta hipoproteica para pacientes com DRC em tratamento conservador por seis meses foi capaz de melhorar alguns fatores de risco cardiovascular, como o excesso de peso e o perfil lipídico plasmático, sugerindo que a dieta hipoproteica, além de outros benefícios pode também ser importante ferramenta para a proteção de doenças cardiovasculares nesses pacientes.

## INTRODUCTION

Chronic kidney disease (CKD) has become a significant public healthcare issue on account of its elevated prevalence and the increased levels of morbidity and mortality the disease introduces in the lives of affected individuals.^1^^,^ 2 ^,^ 3

Early diagnosis of the condition and prompt referral to a service equipped to offer the medical and nutritional care patients need not only delays the progression of renal disease, but also helps treat associated complications and cardiovascular disease (CVD) in paticular.^2^^,^ 4

In the stages of CKD preceding the start of renal replacement therapy, low-protein diet (0.6g/kg/day) becomes an extremely important therapeutic strategy, since it delays kidney failure, improves uremic symptoms, decreases serum phosphorus levels and proteinuria, and improves metabolic acidosis and insulin resistance.^5^^-^ 10

Restricting the ingestion of animal protein decreases the intake of saturated fat and cholesterol, two elements closely associated with the development of dyslipidemia and cardiovascular disease, particularly atherosclerosis.^11^^,^ 12 Obesity and dyslipidemia are often seen in individuals with CKD and may improve with adequate nutritional care.^12^^-^ 16

Therefore, considering the negative impact of cardiovascular risk factors on patients with CKD, particularly in the form of overweight and dyslipidemia, the prescription of low-protein diets to patients managed conservatively may help decrease the weight of overweight and obese patients and improve their lipid profile. However, only a few studies have examined the effects of protein restriction in the modulation of the previously discussed cardiovascular risk factors. Therefore, this study aimed to assess the effects of a low-protein diet on the anthropometric and lipid profile parameters of patients with CKD managed conservatively.

## METHODS

### PARTICIPANTS

This longitudinal clinical trial enrolled 50 patients with CKD stages 3 and 4 referred to the Renal Nutrition Outpatient Unit of the School of Nutrition at Universidade Federal Fluminense, Niterói, RJ, Brazil. Ten patients were lost during the study for giving up participating in the study or missing anthropometric evaluation appointments or collections of biological material. The included patients were 18 years or older, had not been offered nutritional care before, and had glomerular filtration rates ranging from 15 to 44 mL/min/1.73m^2^. Smokers, pregnant patients, patients with autoimmune diseases, neoplasms, liver diseases or AIDS were excluded from the study.

Prior to the start of the study, the patients were informed of the need to collect biological material and signed an informed consent term. The Research Ethics Committee of the School of Medicine-UFF approved the study and issued permit no. 565.857.

### EXPERIMENTAL DESIGN

In the first appointment the patients were prescribed a low-protein (0.6 g of protein /kg of ideal body weight/day) low-salt (5g/day) diet individualized for potassium and phosphorus content. The calculated energy intake was based on the individual nutritional assessment of each patient (30 to 35 kcal/kg of ideal body weight/day). Biochemical, anthropometric, and dietary analyses were performed before and six months after the start of the nutritional intervention. Nutritional follow-up appointments were held every two months and, when needed, adjustments were made to the prescribed diet based on weight oscillations and individual preferences.

### FOOD INTAKE ASSESSMENT

Food intake was assessed before and six months after the start of the nutritional intervention using a 24-hour dietary recall (24HDR) form filled up on one business day and on one weekend day each week for the duration of the study. Energy, macronutrient, and micronutrient intake was estimated with the aid of software program Excel (2010), according to data derived from the Brazilian Food Composition Table (TACO).^17^

### CULINARY WORKSHOPS

Culinary workshops - a theoretical-experiential pedagogical strategy based on the sharing of experiences and participation - were organized to enhance diet compliance. The workshops were designed to develop the culinary skills of the participants, discuss and learn the fundamentals about sources of protein, salt, potassium, and phosphorus, and the proper quantities of these elements in the diets of individuals with CKD. In addition to the discussions held with the group, the participants filled individual written assessment forms, in which they were encouraged to freely record their impressions about the activities they were involved in and the contents presented to them, and to reflect on how the experience might contribute to their daily care.^18^

### NUTRITIONAL STATUS AND BODY COMPOSITION ASSESSMENT

The nutritional status of the patients was assessed by a group of nutritionists in each of the appointments. The patients had their body weight, height, and waist circumference (WC) measured. Nutritional status was assessed based on the BMI, calculated as a ratio between body weight (kg) and height (m) to the square and categorized in accordance with the guidelines set out by the World Health Organization (WHO, 2000).^19^ The values for WC (cm) were compared to the thresholds associated with risk of CVD proposed by the WHO (2000).

Body fat (BFP) and lean mass (LMP) percentages were calculated based on dual-energy X-ray absorptiometry (DXA, Lunar Prodigy Advance Plus, General Electric Madison, Wisconsin, USA) at the Nutritional Assessment Laboratory at UFF (LANUFF) before and six months after the start of the nutritional intervention. The reference values for BFP published Lohman *et al*. (1991)^20^ were used in the study.

### ROUTINE BIOCHEMICAL PARAMETERS AND LIPID PROFILES

Blood samples taken after the patients had fasted for 12 hours were collected before and six months after the start of the nutritional intervention. The serum levels of urea, creatinine, albumin, calcium, potassium, phosphorus, and glucose, and lipid profiles were analyzed on a BioClin^®^ device equipped with the appropriate commercial kits. The GFR was calculated based on the CKD-EPI equation (Levey *et al*., 2009).^21^ Lipid profile parameters - total cholesterol (TC), HDL-cholesterol (HDL-c), and triglycerides (TG) - were analyzed using a KATAL^®^ colorimetric enzymatic kit. The values for LDL-cholesterol (LDL-c) and VLDL-cholesterol (VLDL-c) were calculated using the Friedewald equation. Since there is no consensus over lipid profile target values for patients with CKD - the guidelines from the National Kidney Foundation-Kidney Disease Outcomes Quality Initiative (NKF-KDOQI)^22^ disregard LDL values in treatment decision-making - the lipid profile values found in this study were assessed based on the 5^th^ Brazilian Guidelines for Dyslipidemia and Prevention of Atherosclerosis issued by the Department of Atherosclerosis of the Brazilian Society of Cardiology in 2013 (normal values [mg/dL]): TC < 200, HDL-c > 40, LDL-c < 130, VLDL-c < 30, TG < 150).^23^

### STATISTICAL ANALYSIS

The Kolmogorov-Smirnov test was used to verify the distribution of the variables, with results expressed in mean values ± SD (standard deviation) or proportions, as seen fit. The paired sample t-test, the chi-squared test, and Wilcoxon's test were used to assess the differences resulting from the intervention in the variables of interest. Pearson's correlation coefficient was used to assess the correlations between variables. The tests were set with a confidence interval of 95% and differences with *p* < 0.05 were deemed statistically significant. Statistical analysis was performed on the Statistical Package for Social Sciences version 23.0 (SPSS, Inc., Chicago, IL, USA).

## RESULTS

Half (50%) of the 40 patients enrolled in the study were males. Patient mean age was 62.7 ± 15.2 years, and the mean GFR was 26.2 ± 9.4 mL/min/1.73m^2^. All patients were hypertensive, 45% had diabetes mellitus, and 35% had dyslipidemia and were on lipid-lowering drugs. All patients were on antihypertensive drugs; 20% were on sodium bicarbonate; 32.5% on anti-diabetic medication; 17.5% on antianemia drugs; and 17.5% on vitamin supplements. The use of medication remained unaltered throughout the follow-up period.

Food intake assessment revealed a significant decrease in the intake of energy, protein, cholesterol, and fiber and an increase in the intake of carbohydrates six months after the start of the intervention, as seen on Table 1.

**Table 1:** Dietary parameters of patients with chronic kidney disease managed conservatively at baseline and six months after the start of a low-protein diet

Parameters	Baseline	After 6 months	*p* value
Energy (kcal/kg)	29.0 ± 9.0	23.5 ± 6.4	0.0001
Protein (g/kg)	1.4 ± 0.4	0.8 ± 0.4	0.0001
Carbohydrates (%)	58.1 ± 8.7	62.6 ± 9.0	0.03
Total lipids (%)	21.9 ± 7.9	21.6 ± 9.2	0.83
Monounsaturated fatty acids (%)	31.7 ± 5.4	36.3 ± 14.8	0.11
Polyunsaturated fatty acids (%)	17.7 ± 8.5	15.7 ± 6.4	0.23
Saturated fatty acids (%)	36.8 ± 10.5	33.6 ± 9.9	0.09
Cholesterol (mg)	201.9 ± 92.4	106.0 ± 73.5	0.0001
Fiber (g)	25.2 ± 9.0	22.1 ± 9.6	0.03
Iron (mg)	12.7 ± 26.5	4.7 ± 2.0	0.12

Student’s *t*-test. Differences with *p* < 0.05 were considered statistically significant. Data presented as mean value ± SD. N = 40.

The assessment of anthropometric indicators showed that the BMI had decreased significantly, while WC decreased significantly only among females. BFP and LMP were not significantly different (Table 2).

**Table 2:** Anthropometric profiles of patients with CKD managed conservatively before and six months after the start of a low-protein diet

Parameters	Baseline	After 6 m	*p* value
Weight (kg)	72.9 ± 14.5	70.4 ± 13.4	0.0001
BMI (kg/m^2^)	28.1 ± 5.6	27.0 ± 5.3	0.001
WC (cm) - Females	95.1 ± 15.9	93.2 ± 15.7	0.04
WC (cm) - Males	94.4 ± 14.1	93.1 ± 13.56	0.13
BFP	33.7 ± 8.2	33.4 ± 9.4	0.51
LMP	64.0 ± 5.6	64.6 ± 6.3	0.47

Student’s *t*-test. Differences with *p* < 0.05 were considered statistically significant. N = 40. Data presented as mean value ± SD. BMI = body mass index; WC: waist corcumference; BFP: body fat percentage; LMP: lean mass percentage.

Renal function estimated by the GFR improved six months into the nutritional intervention, while serum uric acid levels decreased (Table 3). Twelve patients (30%) had high serum potassium levels and 14 patients (35%) had serum albumin levels below 3.8 mg/dL at baseline; no improvement was seen after the nutritional intervention (data not shown).

**Table 3:** Biochemical parameters and estimated glomerular filtration rate of patients with CKD managed conservatively six months after the start of a low-protein diet

Parameters	Baseline	After 6 m	*p* value
Urea (mg/dL)	85.2 ± 28.4	79.1 ± 26.4	0.20
Creatinine (mg/dL)	2.6 ± 0.9	2.5 ± 1.2	0.32
GFR (ml/min/1.73m^2^)	26.2 ± 9.5	28.9 ± 12.7	0.02
Uric acid (mg/dL)	6.8 ± 1.4	6.2 ± 1.3	0.004
Glucose (mg/dL)	110.4 ± 42.1	106.9 ± 43.2	0.69
Na (mg/dL)	139.3 ± 3.7	139.4 ± 5.7	0.89
Potassium (mmol/L)	4.8 ± 0.6	4.8 ± 0.5	0.83
Ca (mg/dL)	8.7 ± 1.1	8.9 ± 0.6	0.48
Phosphorus (mg/dL)	3.5 ± 0.7	3.5 ± 0.6	0.99
Iron (µg/dL)	79.3 ± 38.0	81.1 ± 36.9	0.80
Ferritin (µg/dL)	151.4 ± 111.3	128.1 ± 90.8	0.09
Albumin (g/dL)	3.8 ± 0.4	3.8 ± 0.4	0.31

Student’s *t*-test. Differences with *p* < 0.05 were considered statistically significant. N = 40. Data presented as mean value ± SD. GFR: glomerular filtration rate

In terms of lipid profile, 40% of the patients had hypercholesterolemia. The proportion dropped to 27.5% (*p* = 0,01) after the intervention. A significant reduction was seen in the serum levels of TC and LDL-c after six months of low-protein diet; no significant difference was observed in the other parameters (Table 4).

**Table 4:** Lipid profile parameters of patients with CKD managed conservatively six months after the start of a low-protein diet

Parameters (mg/dL)	Baseline	After 6 months	*p* value
Total cholesterol	199.7 ± 57.1	176.0 ± 43.6	0.0001
LDL-c	116.2 ± 48.1	97.4 ± 39.1	0.0001
HDL-c	50.2 ± 14.4	48.7 ± 12.9	0.29
VLDL-c	32.9 ± 13.8	29.4 ± 14.6	0.15
Triglycerides	167.0 ± 71.1	149.7 ± 75.9	0.18

Student's *t*-test. Differences with *p* < 0.05 were considered statistically significant. N = 40. Data presented as mean value ± SD. GFR: glomerular filtration rate.

## DISCUSSION

The low-protein diet prescribed for six months to patients with pre-dialysis CKD in this study preserved renal function, aided in weight loss, and decreased serum levels of uric acid, total cholesterol, and LDL-c.

Since the BMI cannot be used alone to assess body composition, as it does not differentiate muscle mass from fat mass, the BFP and WC have been used as adjuvant methods to assess body fat distribution.^24^^-^ 26 WC has been the method of choice among researchers on account of it low cost and practicality, in addition to its ability to reflect the accumulation of abdominal fat, known for being metabolically active and associated with inflammation and increased risk of death and cardiovascular mortality in particular.^27^

Therefore, despite the significant improvement seen in the anthropometric profiles of the patients after nutritional intervention, no significant improvements were seen in the BFP and LMP. In clinical terms, after the intervention with low-protein diet the patients still were at risk for metabolic syndrome and CVD, since they still were overweight according to the BMI and had abdominal obesity shown by elevated WC measurements.

Obesity has grown significantly within recent decades along with its impact on the onset of noncommunicable diseases (NCDs), including CKD and CVD, and mortality.^28^^-^ 31 A study enrolling Japanese individuals showed that obesity without metabolic anomalies was associated with increased risk of CKD in males, but not in females.^32^ Therefore, therapeutic nutritional measures are needed to reduce the negative impacts of obesity and aid in the treatment of CKD by decreasing proteinuria and glomerular hyperfiltration, improving blood pressure levels, dyslipidemia, insulin resistance, and inflammation.^33^^-^ 35

A study by Lai *et al.* (2015)^36^ included 16 patients with CKD stages 3 and 4 submitted to a low-protein diet for 12 months. Body composition analysis revealed non-significant decreases in body fat and the BMI, and maintenance of lean mass percentages. No decrease was observed in the serum levels of albumin or protein, and kidney function remained stable with significant decreases in urinary protein levels. Non-significant improvements were seen in lipid profiles with decreases in TC, LDL-c, and triglyceride levels, and increases in HDL-c levels. Markers of atherosclerosis and endothelial dysfunction also remained stable.

By their turn, Noce *et al.* (2016)^37^ did not report good results after prescribing a low-protein diet to 41 patients with CKD stages 3b and 4. Although CKD progression was delayed with significant decreases in creatinine and azotemia, the nutritional status of the enrolled patients worsened, with significant decreases in serum albumin and increases in C-reactive protein levels, followed by deterioration of lean mass percentages and elevation of the extracellular mass/body cell mass ratio. Decreases in the phase angle - a negative prognostic factor for survival - were also observed.

In our study, the prescribed low-protein diet improved the GFR, as reported in other studies. The treatment of patients with CKD must consider, among other factors, the rate at which glomerular filtration decreases, along with complications and comorbidities - cardiovascular ones in particular.^38^ A longitudinal study enrolled 239,832 Chinese individuals to examine the association between GFR and CVD and found that patients with lower GFR were at greater risk of obesity, *diabetes mellitus*, hypertension, and dyslipidemia; and at significantly greater risk for coronary artery disease and atherosclerotic cardiovascular disease.^39^

The risks associated with obesity also involve alterations to the lipid profile - or dyslipidemia - caused by impaired lipoprotein catabolism, known as one of the main traditional risk factors for CVD and atherosclerosis in particular.^11^^,^ 40 Dyslipidemia also intensifies the inflammatory process, which ultimately accelerates the progression of CKD.^41^^,^ 42 In addition, CKD impairs the metabolization of lipoproteins.^43^^,^ 44

The decreases in serum levels of total cholesterol and LDL-c reported in this study were expected, since decreased intake of animal protein helps decrease serum cholesterol levels.

In dietary analysis, although percent lipid intake was not significantly reduced, a significant decrease was seen in the intake of dietary cholesterol, thus supporting the results found in lipid profile parameters. The decrease in the daily intake of proteins was statistically significant, and protein intake was positively correlated with dietary cholesterol intake (data not shown), thus supporting the theory that low-protein diets also promote decreases in the intake of dietary cholesterol.

Lipid profile anomalies vary depending on urinary protein levels and stage of CKD. These patients have increased serum triglyceride, decreased HDL-c, increase lipoprotein A, and normal LDL-c levels.^45^ A retrospective cross-sectional study with 136 patients with CKD managed conservatively reported a high prevalence of dyslipidemia (75.7%) and mean values of TC = 179.6 ± 41.0mg/dL, HDL-c = 46.1 ± 12.6mg/dL, LDL-c = 101.7 ± 34.5mg/dL, and triglycerides = 160.0 ± 87.2mg/dL. In addition, the group with dyslipidemia had higher levels of triglycerides and lower levels of HDL-c.^46^

Kanda *et al.* (2016)^45^ showed in a study with 71 patients with DRC stages 4 and 5 that the HDL subclasses were linked to the progression of CKD, suggesting that not only lipoprotein cholesterol levels, but also subclass compositions, might be related to increased risk of death in the population. In another study with 2036 Chinese individuals with a mean GFR of 63 mL/min/1.73m^2^, serum triglyceride levels were negatively correlated with GFR, while the stages of CKD were positively correlated with risk of hypertriglyceridemia.^47^

The prescribed diet included adjustments to energy intake based on the nutritional recommendations provided to the patients in appointments held with nutritionists. The 24HDR forms - a low-cost, quick, practical method - filled up by the patients were used to qualitatively and quantitatively estimate the food intake of the studied population. A limitation inherent to the 24HDR is that it relies on the memory of the individuals recording their meals, which makes it subject to error by under- or overestimation.^48^ In this study the patients reported energy intakes below recommended levels, a finding not supported by the elevated values found for BMI and WC, suggesting food intake was underreported.

Various studies enrolling patients with CKD have reported low levels of compliance to treatment - diet and drug therapy - with numbers ranging from 20% to 70% depending on the evaluation method.^49^ Despite the decrease in protein intake, the mean protein intake six months after the start of the intervention was still above the recommended level. Following a diet rigorously is not easy, since patients are forced to veer away from their eating habits. An additional factor is that in Brazil people eat large amounts of protein in the form of beans, beef, milk, and dairy products,^50^^,^ 51 which makes the compliance to a low-protein diet all the more difficult. Data from the Household Budget Survey of 2009 showed that the most popular food items in the nation were beans, rice, meat, fruit juice (industrialized and fresh), carbonated drinks, and coffee.^52^

In regards to biochemical parameters, only serum uric acid levels were significantly different after the intervention. A possible explanation is the decrease in the intake of foods that increase uric acid levels in the blood, such as red meat, offal, fish, and industrialized and whole foods, among others. This might be seen as a satisfactory outcome, since high uric acid levels have been associated with fast decline of the GFR and increased risk of CKD progression.^53^

The limitations of this study include the small number of enrolled individuals, which limits the consideration of our findings to the studied population; the length of the nutritional intervention, possibly too short to change the eating habits of the participants; and lastly, the use of the 24-hour dietary recall method, which might under- or overestimate food intake. Therefore, more longitudinal studies are required to understand the effects of low-protein diets on the lipid and anthropometric profiles of patients with CKD.

## CONCLUSION

The findings described in this study showed that prescribing a low-protein diet (0.6g/kg/day) for six months to patients with pre-dialysis CKD might delay the progression of CKD and help manage two traditional risk factors for CVD, overweight and dyslipidemia.
